# Depression and Associated Factors among People Living with Human Immunodeficiency Virus Attending Antiretroviral Therapy in Public Health Facilities, Hosanna Town, Southern Ethiopia

**DOI:** 10.1155/2023/7665247

**Published:** 2023-07-25

**Authors:** Markos Hankebo, Chaltu Fikru, Lire Lemma, Gezehagn Aregago

**Affiliations:** ^1^School of Public Health, College of Medicine and Health Sciences, Jimma University, Ethiopia; ^2^Department of Public Health, College of Medicine and Health Sciences, Wachemo University, Southern Nations, Nationalities, and Peoples' Region, Ethiopia; ^3^Nigist Eleni Mohammed Memorial Comprehensive Specialized Hospital, Wachemo University, Southern Nations, Nationalities, and Peoples' Region, Ethiopia

## Abstract

**Background:**

Among those infected with the human immunodeficiency virus, depression is one of the most prevalent mental health issues. Despite its high incidence, depression goes undiagnosed and untreated in the majority of HIV/AIDS patients, which has a negative impact on how well they adhere to their antiretroviral regimen.

**Objective:**

To assess the magnitude of depression and associated factors among people attending antiretroviral therapy in public health facilities of Hosanna town, Hadiya Zone, Southern Ethiopia, 2019.

**Methods:**

Institution-based cross-sectional study was conducted at public health facilities of Hosanna town from June 6 to July 6, 2019, among people living with HIV/AIDS aged 18 years and older who were on ART. A systematic sampling technique was used to select 392 participants. Data were collected using a pretested and standardized structured interviewer-administered questionnaire. Variables having a *p* value less than 0.2 in bivariate analysis were entered into the multiple logistic regression model. Odds ratio with 95% CI was computed, and variables with *p* value < 0.05 were considered as statistically significantly associated with depression.

**Result:**

The prevalence of depression among HIV patients was 37.8%. Being female (AOR = 2.15, 95% CI (1.21, 3.84)), not disclosing their HIV status (AOR = 2.77, 95% CI (1.57, 4.89)), rural dwellers (AOR = 2.69, 95% CI (1.58, 4.57)), poor ART adherence (AOR = 1.89, 95% CI (1.10, 3.24)), having HIV-perceived stigma (AOR = 1.71, 95% CI (1.01, 2.88)), and poor social support (AOR = 1.85, 95% CI (1.11, 3.09)) were significantly associated with depression.

**Conclusion:**

The magnitude of depression was high among PLWHIVs. Being female, rural dwellers, not disclosing HIV status, poor ART adherence, HIV-perceived stigma, and poor social support were significantly associated with depression. Enhancing adherence, counseling, and linking those patients who had poor social support to the concerned relatives for care and support is recommended. Providing health education both at the facility level and at the community level may reduce stigma and subsequently depression. Encouraging disclosing HIV status may help to prevent depression.

## 1. Introduction

Depression is a common and treatable mental illness marked by a lack of interest and enjoyment, diminished energy, guilt or feelings of low self-worth, interrupted sleep and/or food, and poor attention for more than two weeks [1, 2].

Unknown causes lead to depression. The most probable cause of depression is a confluence of genetic, physiological, environmental, and psychological factors [3, 4].

According to the WHO 2017 report, more than 300 million people are living with depression worldwide [5]. By accounting for 4.3% of all disability-adjusted life years, depression ranks third among the world's top causes of disease burden and is expected to overtake malaria by the year 2020 [6]. In Ethiopia, depression contributes to about 6.5% of the burden of diseases. This is the highest share of burden compared to other forms of mental disorders [7].

Infection with the human immunodeficiency virus (HIV) has emerged as a genuine societal emergency [8]. HIV infection and depression have a complicated relationship in which depression may occur before the diagnosis and be linked to risk factors for HIV infection; yet, having HIV/AIDS may make depressive episodes worse [9].

One of the most prevalent mental health conditions among those living with HIV/AIDS is depression. Compared to most other chronic illnesses, it results in more disability and larger health declines [9, 10]. The prevalence of depression among PLWHIVs varied from 20 to 48% in high-income nations and from 9 to 72% in nations with scarce resources [11]. According to estimates, between 29 and 63.1% of PLHIV on antiretroviral therapy in sub-Saharan Africa suffer from depression [12], and the pooled prevalence of depression in PLWHIV in Ethiopia was 36.65% [13]. One in three PLHIV individuals may experience depression. Although depression is common among those living with HIV, it is typically an undiagnosed and untreated illness [11].

The prevalence of depressive disorders among PLHIVs worldwide is considerable, and they have a detrimental effect on ART adherence [11]. Depressed HIV patients had a weaker immune function, lower antiretroviral medication (ART) adherence, slower viral suppression, faster development to AIDS, and higher mortality risk compared to those without depression [14, 15].

The presence of depression in PLHIV causes changes in economic output, a drop in working capacities, social isolation, physical decline, and challenges with problem-solving. The risk of strain on the health care systems and human resources is high as depression among PLHIV arises as a public health issue, particularly in sub-Saharan Africa [16]. Each year, the cost of medical care for depression exceeds $43 billion, and the cost of lost productivity is over $17 billion [17].

Low CD4 counts, being female, uneducated, divorced or widowed, being older than 40, being unemployed, abusing drugs or alcohol, not taking their medications as prescribed, being in the early stages of HIV infection, feeling stigmatized, and having little social support were all factors that were linked to depression in HIV-infected patients [8, 12, 13]. Even though some studies were conducted on depression and associated factors among HIV patients, there is wide variation in the prevalence of depressive disorders across studies. Moreover, the inclusion and exclusion criteria of participants vary among these studies. Some studies used a small sample [18, 19]. In addition to these, as per my knowledge, no study was conducted on depression and associated factors among PLHIVs in the study area. Hence, this study assessed the prevalence of depression among PLWHAs and associated factors in Hosanna town, Hadiya Zone, Southern Ethiopia.

## 2. Methods

### 2.1. Study Area and Period

The study was conducted in public health facilities of Hosanna town, Hadiya Zone, from June 6 to July 6, 2019. Hosanna town is located 232 km southwest of Addis Ababa, the capital of Ethiopia [20].

The town has one comprehensive specialized hospital, one private hospital, and three public health centers. Two of them provide ART services to the clients, and this study was conducted in two of the health facilities, namely, Nigist Eleni Mohammed Memorial Comprehensive Specialized Hospital and Hosanna Health Center.

There were 770 PLWHIVs on ART follow-up in Nigist Eleni Mohammed Memorial Comprehensive Specialized Hospital and 110 in Hosanna Health Center [21].

### 2.2. Study Design

An institution-based cross-sectional study was conducted.

### 2.3. Source Population

The source population was all PLWHIV attending ART at Nigist Eleni Mohammed Memorial Comprehensive Specialized Hospital and Hosanna Health Center.

### 2.4. Study Population

The study population was all PLWHIV aged 18 years and above attending ART during the data collection period.

### 2.5. Inclusion and Exclusion Criteria

Inclusion criteria are as follows: PLHIV on HAART for at least the past 6 months and aged 18 years and above.

Exclusion criteria are as follows: patients who had lost a loved one within the last 3 months were excluded because they are likely to present with post-traumatic depression. And acutely ill patients were also excluded because they cannot be stable and respond to the interview appropriately.

### 2.6. Sample Size and Sampling Procedure

Single population proportion formula was used to calculate the sample size for the first specific objective considering the following assumptions:

(1) $$\begin{array}{c} n={\left(\frac{\text{z}\alpha }{2}\right)}^{2}\;\frac{p\;\left(1-p\right)}{d^{2}}=357 \end{array}$$

where *p* takes the prevalence of depression among PLWHAs (36.65%) [13], *d* is the margin of error (0.05), and *zα*/2 = 1.96 is the *z*-score corresponding to a 95% confidence level.

The sample size for the second specific objective was determined by using Epi Info version 7.

Assumptions to calculate sample size are as follows: 95% confidence interval (95% CI), power=80%, and ratio of exposed to nonexposed=1.

The outcome in unexposed is the magnitude of depression among PLHIVs who are not with perceived stigma, have good social support, and are in WHO clinical stage I.

The outcome in exposed is the magnitude of depression among PLHIVs who are with HIV-perceived stigma, have poor social support, and are in WHO clinical stage II (Table 1).

**Figure 1:**
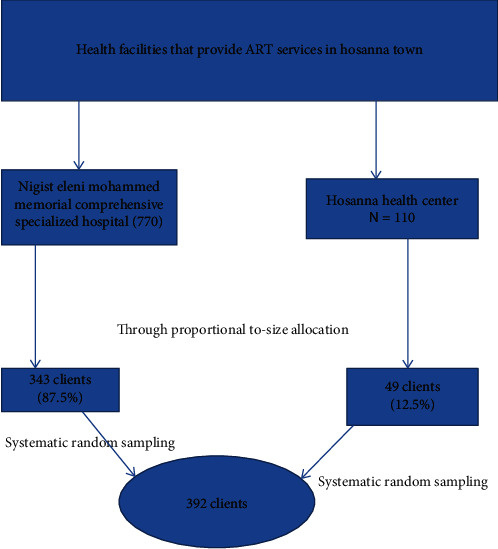
Schematic presentation of the sampling procedure.

Taking the largest sample size of 357 and considering 10% of the nonresponse rate, the total sample size of 392 was considered. Then, this sample size was allocated to two health facilities, namely, Nigist Eleni Mohammed Memorial Comprehensive Specialized Hospital and Hosanna Health Center based on the proportional to size allocation method. Accordingly, 343 (87.5%) of the sample were from Nigist Eleni Mohammed Memorial Comprehensive Specialized Hospital, and 49 (12.5%) of the sample were from Hosanna Health Center. Systematic random sampling was used to select study participants from each public health facility. The sampling interval was 2. Whenever the sampled client did not fulfill the inclusion criteria, immediately the next one, who was eligible, was interviewed (Figure 1).

### 2.7. Data Collection Tool and Procedure

A standardized structured interviewer-administered questionnaire was adopted from PHQ-9 (Patient Health Questionnaire-9) [23], Ethiopian NCD STEPS survey questionnaire, Oslo 3-item social support scale (OSSS-3), self-report measure of support, national guidelines for comprehensive HIV prevention, care and treatment, and Berger's Revised Stigma Scale. These tools were also supplemented by patient medical record reviews. A structured questionnaire was developed by reviewing different literature and used to collect data on sociodemographic and economic characteristics. A checklist was used to collect data on clinical characteristics. The questionnaire was translated from the English language into the Hadiyisa language and backtranslated to the English language by different personnel for the sake of consistency. Four diploma nurses have collected and two health officers have supervised data collection daily. The data were collected using an interview technique in a private room after the client gets service at ART clinics.

### 2.8. Study Variables and Measurement

Dependent variable is categorised as follows: depression (yes/no).

Independent variables are as follows:

Sociodemographic and economic factors: age, sex, educational status, residence, marital status, income, ethnicity, religion, occupation, and living condition.

Clinical factors: CD4 count, comorbidities, WHO clinical stage, ART adherence, and type of ART regimen.

Psychosocial factors: HIV-perceived stigma, social support, and substance use including the use of chat, alcohol, and cigarette for non-medical purposes.

PHQ-9 (Patient Health Questionnaire-9) was used to measure the depression level among PLWHIVs. It contains nine items that measure the frequency of depressive symptoms over the past 2 weeks [23]. The response options for each item are “not at all,” “on several days,” “on more than half of the day,” and “nearly every day,” which are scored 0–3. The sum of the scores can range from 0–27, with the higher scores indicating more severe depression [24]. Regarding severity, PHQ-9 comprises five categories, where a cut-off point of 0–4 indicates no depressive symptoms, 5–9 mild depressive symptoms, 10–14 moderate depressive symptoms, 15–19 moderately severe depressive symptoms, and 20–27 severe depressive symptoms. PHQ-9 items showed good internal (Cronbach's alpha = 0.85) and test retest reliability (interclass correlation coefficient = 0.92). It is commonly used to screen for symptoms of depression in primary health care and outpatients and validated in Ethiopia with sensitivity = 86% and specificity = 67% [25].

Substance use was assessed using an adapted Ethiopian NCD STEPS survey questionnaire [26].

ART adherence was assessed using tools adopted from national guidelines for comprehensive HIV prevention, care, and treatment [27].

Social support was measured using the Oslo 3-item social support scale (OSSS-3) and self-report measure of support with internal consistency (Cranach's *α* = 0.64) and good validity [28, 29].

HIV-perceived stigma was assessed by Berger's Revised Stigma Scale 10 items which measure perceived stigma in PLWHIV using a 5-point Likert scale from “strongly disagree” to “strongly agree,” with items categorized into four dimensions: personalized stigma, disclosure concerns, negative self-image, and concerns with public attitudes [30, 31].

### 2.9. Operational Definitions

Depressed: participants who score 5 and above on self-reporting questionnaire (PHQ-9) [15].

Nondepressed: participants who score below 5 on SRQ (PHQ-9).

Comorbid diseases: proven or diagnosed medical problems in addition to the existing HIV/AIDS [32]. It will be proven by reviewing the patient chart and asking the patient.

HIV-perceived stigma: it was assessed by taking a mean of the total from a rate 0-20 as not perceived stigma and 21-40 as had perceived stigma [32].

Substance: alcohol, chat, and tobacco products like cigarettes and chewing tobacco.

Substance ever used: using a specific substance at least once in a lifetime (nonmedical use) [33].

Current substance use: using a specific substance within 30 days before the study (nonmedical use) [33].

Poor social support is defined as when an individual scores “3-8” based on the Oslo 3-item Social Support Scale [29].

Moderate social support is defined as when individual scores “9-11” based on Oslo 3-item Social Support Scale.

Strong social support is defined as when an individual scores “12-14” based on the Oslo 3-item Social Support Scale.

Medication adherence refers to compliance with the patient's behavior in taking ARV medication [27].

Good = client misses <2 doses from 30 doses or <3 from 60 doses of ARV.

Fair = client misses 3-4 doses from 30 doses or 4-9 doses from 60 doses of ARV.

Poor = client misses >5 doses from 30 doses or >9 doses of ARV.

### 2.10. Data Analysis Procedures

Data were checked, coded, and entered into EpiData 3.1 and exported to Stata version 15 for analysis. The extent of the outlier was checked using a box plot and no outlier was found, and missing values were checked. Tables and charts were used to present the results of the study. Binary logistic regression was used for analysis. Bivariate binary logistic regression analysis was done to see the association of each independent variable with the outcome variable. Those variables having a *p* value less than 0.2 were entered into the multiple binary logistic regression model to identify the independent effect of independent variables with the outcome variables and to control potential confounders. The odds ratio with 95% CI was calculated to assess the association between independent and outcome variables. The model fitness was checked by the Hosmer-Lemeshow goodness of fit test. Variables with a *p* value of less than 0.05 were considered significantly associated in the final model.

### 2.11. Data Quality Management

To assure the data quality, training was given to the data collectors and supervisors on objectives, data collection methods, tools, and how to handle ethical issues. The questionnaire was pretested among 5% of the total samples in Morsito Health Center, one of the health centers located in the Hadiya Zone which gives care and treatment for patients with HIV/AIDS. Based on the result of the pretest, necessary changes like reordering the questions and replacing some words with similar Hadiyisa words were made on the data collection tools.

## 3. Results

### 3.1. Sociodemographic and Economic Characteristics of Participants

A total of 392 study participants were included in the study with a 100% response rate. The mean age of the respondents was 34.02 (±SD = 7.603) years. More than half of the participants (57%) were female, 219 (55.9%) were married, and 101 (25.7%) were self-employed (Table 2) [29].

### 3.2. Clinical, Psychosocial, and Substance Use Characteristics of the Respondents

Regarding clinical characteristics of study participants, 312 (79.6%)) were on ART for greater than 12 months. Around half (50.8%) of respondents had a CD4 count less than or equal to 500 cells/*μ*L. More than three-fourths of study participants (79.3%) were in WHO clinical stage I. More than half (52.6) of the participants had good ART adherence, 209 (53.3%) had poor social support, 244 (62.2%) had HIV-perceived stigma, 64 (16.3%) had comorbid tuberculosis, and 255 (65.1%) did not disclose their HIV status other than health care provider (Table 3).

### 3.3. Prevalence of Depression among People Living with HIV/AIDS

**Figure 2:**
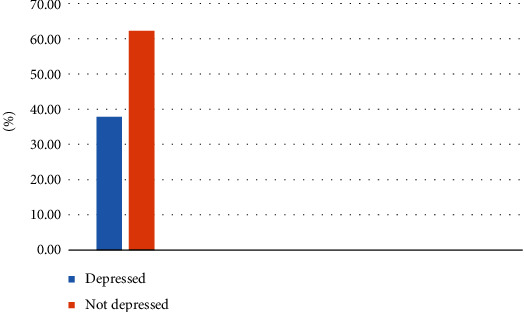
Magnitude of depression among people living with HIV at public health facilities in Hosanna town, Hadiya Zone, Southern Ethiopia, 2019.

The prevalence of depression among PLHIV was 37.8% (95% CI: 33.2, 42.9) (Figure 2). Of which 139 (35.5%) were at Nigist Eleni Mohammed Memorial Comprehensive Specialized Hospital and 9 (2.3%) were at Hosanna Health Center. The prevalence of depression was 30.95% (52) among males and 44.82% (96) among females. The prevalence of depression was 33.34%, 24.65%, 62.68%, and 63.63% among single, married, divorced, and widowed, respectively (Table 4).

### 3.4. Factors Associated with Depression among People Living with HIV

In bivariate analysis, variables such as sex, residence, age, educational status, marital status, occupation, living condition, ART adherence, social support, HIV-perceived stigma, and WHO clinical stage of HIV had *p* values < 0.2, and they were considered for multivariable binary logistic regression.

In a multivariable binary logistic regression, social support, ART adherence, HIV-perceived stigma, disclosing HIV status, place of residence, and sex were significantly associated with depression among PLWHIVs.

Those who had poor social support had 1.85 times higher odds of depression than those who had good social support (AOR = 1.85, 95% CI: 1.11, 3.09). Those PLWHIVs who had poor ART adherence had 1.89 times higher odds of depression as compared to those who had good adherence (AOR = 1.89, 95% CI: 1.10, 3.24). Participants who had HIV-perceived stigma had 1.71 times higher odds of depression as compared to those who had no perceived stigma (AOR = 1.71, 95% CI: 1.01, 2.88). Those PLWHIVs who were not disclosing HIV status to someone other than health care providers had 2.77 times higher odds of depression as compared to those who were disclosing HIV status (AOR = 2.77, 95% CI: 1.57, 4.89). Rural dwellers had 2.69 times higher odds of depression compared to urban dwellers (AOR = 2.69, 95% CI: 1.58, 4.57). Females had 2.15 times higher odds of depression as compared to males (AOR = 2.15, 95% CI: 1.21, 3.84) (Table 4).

## 4. Discussion

A high magnitude of depression is noted (37.8%, 95% CI: 33.2, 42.90). This conclusion is consistent with research done at Debre Berhan Referral Hospital (38.94%), in three regions of Ethiopia, Nigeria, and Brazil, where prevalence estimates were found to be 36.65, 41.2%, 39.6%, and 42.3%, respectively [8, 9, 12, 13, 22]. The prevalence in this study was higher than that in studies done in various parts of Ethiopia, Myanmar, and Cameroon, where it was reported to be 11.7%, 14.6%, 32.9%, 26.7%, and 30.12%, respectively [19, 32, 34, 35]. However, the results of the current study were lower than those of studies conducted in two regions of India, Delhi, Brazil, and Sudan, where the prevalence was reported to be 50%, 55.6%, 58.75%, 53.5%, and 63.1%, respectively [36–40]. The range in the prevalence of depression among studies may be explained by differences in sample size, the method used to measure depression, and sociodemographic diversity.

Being female, not disclosing HIV status, living in rural areas, perceived HIV stigma, poor social support, and poor ART adherence were significantly associated with depression.

When compared to individuals who had good ART adherence, those with poor ART adherence were 1.89 times more likely to experience depression. Studies carried out in various parts of Ethiopia provided evidence in support of this [10, 22, 32]. An increased risk of poor adherence correlated with the higher prevalence of depression symptoms and depression may also induce poor adherence among PLWHIVs. Skipping HIV medicines allows HIV to multiply, which increases the risk of drug resistance and other complications like depression and opportunistic diseases [35]. Similarly, poor social support was linked to an increased risk of depression. Comparing those with good social support to those with low social support, depression risk was greater for those with poor social support. This is consistent with research from Alert Hospital in Ethiopia, Delhi, and the Brazilian Amazon [22, 39]. Due to social stigma against oneself, HIV/AIDS patients chose to avoid asking for aid and disclosing their health, which increased their loneliness and isolation [22].

When compared to HIV patients who were not stigmatized, those who felt stigma had a higher likelihood of developing depression. Studies conducted in Ethiopia at the Debre Berhan Hospital, Alert Hospital, Zewditu Memorial Hospital, and Debre Markos Hospital provided evidence in support of this [9, 15, 19, 22]. The possibility of being ignored by others, losing their job, and resulting in financial difficulties could be the cause. In this study, keeping one's HIV status a secret was linked to depression. In contrast to those who disclosed their HIV status, those who did not have increased odds of developing depression. This is consistent with a Malawian study [41]. This might be because people who did not reveal their HIV status might not use ART effectively, which results in poor adherence and may cause depression. Females had higher odds of depression compared to males. This is supported by studies done in different parts of the world. Women experience higher levels of major depression in comparison to their male counterparts as evidenced in many studies. This pattern continues in people with HIV, with multiple studies showing a higher magnitude of depression in women with HIV than men with HIV; the biological or hormonal difference between females and males might explain the higher odds of depression among females than males [9, 12, 16, 38, 42, 43]. In contrast to this finding, a study done in Harar reported that male PLWHIVs were more depressed than female [10]. Compared to people living in urban, those who lived in rural areas had an increased probability of developing depression. This may be because PLWHIV in rural areas has limited access to information on HIV/AIDS and ways to lessen the perceived stigma associated with the disease.

The limitation of the study is as follows: the use of nurses who work at the ART clinic of each health facility as data collectors may induce a social desirability bias. The use of self-reported information may be subjected to reporting errors.

## 5. Conclusion

The prevalence of depression was high (37.8%) among PLWHIVs, and depression was significantly associated with being female, rural dwellers, not disclosing HIV status, having poor ART adherence, HIV-perceived stigma, and poor social support. Enhancing adherence, counseling, and linking those patients who had poor social support to the concerned relatives for care and support is recommended. Providing health education both at the facility level and at the community level may reduce stigma and subsequently depression. Encouraging disclosing HIV status may help to prevent depression.

## Figures and Tables

**Table 1:** Sample size calculation for the second specific objective.

References	Associated factors	Assumptions	Sample size
[15]	Perceived HIV stigma	95% CI, OR = 2.21, ratio 1 : 1 Depression among PLHIV not stigmatized is 34.14%	228
[22]	Social support	95% CI, OR = 2.02, ratio 1 : 1 Depression among PLHIV with good social support is 27.32%	308
[9]	WHO clinical stage	95% CI, OR = 2.317, ratio 1 : 1 Depression among PL HIV with WHO clinical stage I is 32.1%	206

**Table 2:** Sociodemographic and economic characteristics of PLWHIV at public health facilities of Hosanna town, Hadiya Zone, Southern Ethiopia, 2019 (*n* = 392).

Variables	Categories	Frequency	Percent (%)
Sex	Male	168	43
	Female	224	57

Age (years)	18-29	116	29.6
	30-39	190	48.5
	≥40	86	21.9

Marital status	Single	51	13
	Married	219	55.9
	Divorced	67	17.1
	Widowed	55	14

Educational status	Unable to read and write	102	26
	Primary	122	31.1
	Secondary	78	19.9
	Diploma and above	90	23

Religion	Protestant	190	48.5
	Orthodox	133	33.9
	Others	69	17.6

Ethnicity	Hadiya	235	59.9
	Gurage	64	16.3
	Kembata	33	8.4
	Amhara	33	8.4
	Others	27	6.9

Occupation	Government employee	58	14.8
	Student	31	7.9
	Housewife	74	18.9
	Daily laborer	54	13.8
	Self-employed	101	25.7
	Unemployed	74	18.9

Residence	Urban	247	63
	Rural	145	37

Living condition	With family members	359	91.6
	Alone	33	8.4

Income (ETB)	≤500	27	6.9
	500-1000	77	19.6
	1001-1500	81	20.7
	≥1500	207	52.8

Hint: income (ETB) classification: adapted from reference (29).

**Table 3:** Distribution of clinical, psychosocial, and substance use characteristics among PLWHIVs at public health facilities of Hosanna town, Hadiya Zone, Southern Ethiopia, 2019 (*n* = 392).

Variables	Categories	Frequency	Percent (%)
Duration on ART	≤12 months	80	20.4
	>12 months	312	79.6

Comorbid disease	Tuberculosis	64	16.3
	Diabetes mellitus	29	7.4
	Hypertension	21	5.4
	Dermatitis	45	11.5
	Others	11	2.8
	No	222	56.6

Stage of HIV/AIDS	Stage 1	311	79.3
	Stage 2	47	12
	Stages 3 and 4	34	8.7

CD4 count	≤500	199	50.8
	>500	193	48.2

ART adherence status	Good	206	52.6
	Poor	186	47.4

Types of ART (regimen)	First line	335	85.5
	Second line	57	14.5

Disclosure of HIV status	Yes	137	34.9
	No	255	65.1

Social support	Good	183	46.7
	Poor	209	53.3

HIV-perceived stigma	Yes	244	62.2
	No	148	37.8

Substance ever used	Yes	35	8.9
	No	357	91.1

Substance current use	Yes	32	8.2
	No	360	91.8

**Table 4:** Bivariable and multivariable analysis for factors associated with depression among PLWHIVs at public health facilities of Hosanna town, Hadiya Zone, Southern Ethiopia, 2019 (*n* = 392).

Explanatory variable	Category	Depression		COR, 95% CI	*p* value	AOR, 95% CI
		Yes	No			
Sex	Male	52	116	1.00	**0.020**	1.00
	Female	96	128	1.67 (1.09, 2.55)		2.15 (1.21, 3.84)^∗^

Age	18-29	32	77	1.00		1.00
	30-39	79	126	0.46 (0.25, 0.84)	**0.042**	1.41 (0.71, 2.79)
	≥40	37	41	0.69 (0.41, 1.18)	**0.045**	0.93 (0.37, 2.31)

Residence	Urban	71	176	1.00	**≤0.001**	1.00
	Rural	77	68	2.81 (1.83, 4.30)		2.69 (1.58, 4.57)^∗^

Educational status	Unable to read and write	53	49	2.06 (1.15, 3.69)	**0.002**	0.94 (0.39, 2.25)
	Primary	45	77	1.11 (0.63, 1.97)	0.02	0.66 (0.28, 1.53)
	Secondary	19	59	0.61 (0.31, 1.20)	0.30	0.59 (0.23, 1.58)
	Diploma and above	31	59	1.00		1.00

Religion	Protestant	65	125	1.00		
	Orthodox	51	82	1.19 (0.76, 1.89)	0.203	
	Others	32	37	1.66 (0.95, 2.91)	0.32	

Ethnicity	Hadiya	91	144	1.00		
	Gurage	24	40	0.95 (0.54, 1.68)	0.799	
	Kembete	10	23	0.69 (0.31, 1.51)	0.25	
	Amhare	11	22	0.79 (0.37, 1.71)	0.27	
	Others	12	15	1.27 (0.57, 2.83)	0.32	

Marital status	Single	17	34	1.00		1.00
	Married	54	165	0.66 (0.34, 1.26)	**0.06**	0.43 (0.16, 1.17)
	Divorced	42	25	3.36 (1.56, 7.21)	**0.002**	1.25 (0.43, 3.96)
	Widowed	35	20	3.50 (1.57, 7.79)	**≤0.001**	2.16 (0.65, 7.17)

Occupation	Government employed	14	44	1.00		1.00
	Student	9	22	1.28 (0.48, 3.43)	0.31	0.73 (0.18, 2.95)
	Housewife	28	46	1.91 (0.89, 4.10)	0.47	1.60 (0.53, 4.87)
	Daily laborer	24	30	2.51 (1.12, 5.63)	**0.02**	1.75 (0.53, 5.74)
	Self-employed	36	65	1.74 (0.84, 3.59)	0.47	1.71 (0.60, 4.88)
	Unemployed	37	37	3.14 (1.48, 6.68)	**0.047**	1.47 (0.49, 4.43)

Living condition	With family members	128	231	1.00	**0.006**	1
	Alone	20	13	2.78 (1.34, 5.77)		2.30 (0.96, 5.51)

Disclose of HIV status	Yes	31	106	1.00	**≤0.001**	1.00
	No	117	138	2.89 (1.81, 4.63)		2.77 (1.57, 4.89)^∗^

Income (ETB)	≤500	12	15	1.5 (0.67, 3.38)	0.583	
	500-1000	30	47	1.19 (0.69, 2.05)	0.25	
	1001-1500	34	47	1.36 (0.80, 2.29)	0.22	
	≥1500	72	135	1.00		

Duration on ART	≤12 months	27	53	0.80 (0.48, 1.35)	0.408	
	>12 months	121	191	1.00		

CD4 count	≤500	71	128	0.84 (0.56, 1.26)	0.389	
	>500	77	116	1.00		

Clinical stage of HIV	Stage 1	109	202	1.00		1
	Stage 2	19	28	1.26 (0.67, 2.36)	0.23	0.67 (0.31, 1.48)
	Stages 3 and 4	20	14	2.65 (1.29, 5.45)	**0.028**	1.46 (0.59, 3.61)

Comorbid disease	Yes	69	101	1.24 (0.82, 1.87)	0.312	
	No	79	143	1.00	0.80	

Type of ART regimen	First line	129	209	1.00	0.27	
	Second line	19	37	1.32 (0.35, 2.52)		

ART adherence	Good	57	149	1.00	**≤0.001**	1.00
	Poor	91	95	2.50 (1.64, 3.81)		1.89 (1.10, 3.24)^∗^

Social support	Good	51	133	1.00	**≤0.001**	1.00
	Poor	97	111	2.28 (1.49, 3.48)		1.85 (1.11, 3.09)^∗^

HIV-perceived stigma	No	41	107	1.00	**0.002**	1.71 (1.01, 2.88)^∗^
	Yes	107	137	2.04 (1.31, 3.16)		1.00

Substance ever used	Yes	13	22	0.97 (0.47, 1.99)	0.94	
	No	135	222	1.00		

Substance current use	Yes	11	21	0.85 (0.39, 1.82)	0.68	
	No	137	223	1.00		

Hint: 1-reference category. ^∗^*p* value < 0.05. The bold *p* values show the independent variables with *p* values less than or equal to 0.2 in a bivariate binary logistic regression analysis. 0.2 in a bivariate binary logistic regression analysis is the cut-off point to consider a variable for a multivariable binary logistic regression.

## Data Availability

The datasets used and analyzed during the current study are available from the corresponding author on reasonable request.
